# METTL3‐Mediated m6A Modification of ISG15 mRNA Regulates Doxorubicin‐Induced Endothelial Cell Apoptosis

**DOI:** 10.1111/jcmm.70339

**Published:** 2025-01-09

**Authors:** Dongdong Jian, Han Li, Chenqiu Wang, Fang Li, Runhua Li, Shouyi Jin, Jia Shen, Jiamian Chen, Wanjun Zhang, Ling Pan, Wengong Wang, Hao Tang, Liguo Jian, Datun Qi

**Affiliations:** ^1^ Department of Biochemistry and Molecular Biology, Beijing Key Laboratory of Protein Posttranslational Modifications and Cell Function, School of Basic Medical Sciences Peking University Health Science Center Beijing China; ^2^ Zhengzhou Key Laboratory of Cardiovascular Aging, Henan Province Key Laboratory for Prevention and Treatment of Coronary Heart Disease, National Health Commission key Laboratory of Cardiovascular Regenerative Medicine Central China Fuwai Hospital of Zhengzhou University, Fuwai Central China Cardiovascular Hospital & Central China Branch of National Center for Cardiovascular Diseases Zhengzhou Henan China; ^3^ Department of Cardiology The Second Affiliated Hospital of Zhengzhou University Zhengzhou Henan China; ^4^ Tianjian Laboratory of Advanced Biomedical Sciences, Institute of Advanced Biomedical Sciences Zhengzhou University Zhengzhou Henan China; ^5^ Department of Hematology Henan Provincial People's Hospital Zhengzhou Henan China; ^6^ Nursing Department Haining People's Hospital (Haining Branch, the First Affiliated Hospital, Zhejiang University) Zhejiang China

**Keywords:** apoptosis, doxorubicin, endothelial cell, ISG15, METTL3

## Abstract

N6‐adenosine methylation (m6A) of RNA is involved in the regulation of various diseases. However, its role in chemotherapy‐related vascular endothelial injury has not yet been elucidated. We found that methyltransferase‐like 3 (METTL3) expression was significantly reduced during doxorubicin (DOX)‐induced apoptosis of vascular endothelial cells both in vivo and in vitro, and that silencing of METTL3 further intensified this process. Combined transcriptome and proteome sequencing analyses revealed that the expression levels of interferon‐stimulated gene 15 (ISG15) mRNA and protein significantly increased after METTL3 silencing. Methylated RNA immunoprecipitation (meRIP)‐quantitative polymerase chain reaction (qPCR) and mRNA stability assays confirmed that METTL3 regulates the expression of ISG15 by methylating the 1,014,147 site on ISG15 RNA, thereby decreasing ISG15 mRNA levels. Silencing ISG15 significantly suppressed DOX‐induced endothelial cell apoptosis and dysfunction caused by METTL3 silencing. In summary, our study revealed that METTL3‐mediated methylation of ISG15 mRNA is involved in DOX‐induced endothelial cell apoptosis and explored potential therapeutic targets for alleviating chemotherapy‐associated vascular injury.

## 1 Introduction

The survival rate of cancer patients has significantly improved with the use of various chemotherapeutic drugs. However, the cardiotoxic adverse reactions associated with the use of chemotherapeutic drugs have become increasingly apparent. In addition to limiting the effectiveness of cancer treatment, these adverse reactions also severely affect the quality of life of cancer patients [1]. Chemotherapeutic drugs damage vascular endothelial cells, leading to the disruption of vascular endothelial homeostasis, which is the pathological basis for a series of cardiovascular toxic adverse effects, such as myocardial infarction, hypertension and atherosclerosis [2]. Varying degrees of apoptosis have been observed in the vascular endothelial cells in these pathological conditions [3, 4]. However, the detailed mechanisms underlying the occurrence and development of these conditions have not been fully elucidated. Therefore, studying the regulatory effects of chemotherapy drugs on vascular endothelial apoptosis is expected to be helpful in reducing cardiovascular adverse reactions and improving chemotherapy tolerance during chemotherapy and holds substantial importance in improving the survival rate and quality of life of patients with cancer and enhancing the effectiveness of cancer treatment.

N6‐adenosine methylation (m6A) is widespread in eukaryotic RNA. Previous studies have shown that m6A modification is involved in the occurrence and development of various systemic diseases, including cardiovascular, cancerous, reproductive and nervous system diseases [5, 6]. Methyltransferase‐like 3 (METTL3), an important component of the m6A methyltransferase complex, plays an important regulatory role in various cellular biological processes such as endothelial cell proliferation, ageing and inflammation [7]. In addition, METTL3 has been found to be involved in the apoptosis of various cells, including tumour cells, chondrocytes in inflammation and podocytes in diabetic nephropathy [8, 9, 10]. Previous studies have shown that METTL3‐mediated m6A promotes cell inflammation and apoptosis in paediatric pneumonia models by regulating the expression of the EZH2 protein [11]. Liu et al. confirmed that METTL3‐mediated m6A modification promotes the occurrence and development of experimental osteoarthritis by regulating the inflammatory response and apoptosis of chondrocytes [12]. Our previous study also reported that METTL3/methyltransferase‐like 14 (METTL14) can promote the translation of forkhead box protein O1 (FOXO1) mRNA through RNA methylation, thereby upregulating its expression, activating the transcription of intercellular adhesion molecule 1 (ICAM‐1) and vascular cell adhesion molecule 1 (VCAM‐1) and promoting vascular endothelial inflammation and the formation of atherosclerosis [13]. However, the involvement of METTL3 in the regulation of vascular endothelial cell apoptosis induced by chemotherapeutic drugs, which leads to vascular endothelial cell dysfunction and related cardiovascular complications, has not yet been reported.

In this study, we confirmed that METTL3 regulates the half‐life of interferon‐stimulated gene 15 (ISG15) mRNA by methylation, thereby suppressing interferon‐stimulated gene 15 (ISG15) expression and participating in doxorubicin (DOX)‐induced endothelial cell apoptosis. We further explored the impact of this regulatory effect on the expression of endothelial inflammatory factors and monocyte‐endothelial adhesion, providing new insights for the protection of the endothelium in tumour patients during clinical chemotherapy.

## 2 Materials and Methods

### 2.1 Reagents and Antibodies

Doxorubicin (DOX; HY‐15142) and actinomycin D (HY‐17559) were purchased from MCE and dissolved according to the manufacturer's instructions. Annexin V‐Elab Fluor 647/PI Apoptosis Kit was purchased from Elabscience (E‐CK‐A213). METTL3 rabbit polyclonal antibody (pAb; A8370), ISG15 rabbit monoclonal antibody (mAb; A2416) and Bcl‐2 Rabbit pAb (A0208) were purchased from ABclonal Technology. P53 (sc‐126) antibody was purchased from Santa Cruz Biotechnology (Santa Cruz, CA, USA). Cleaved caspase‐3 (Asp175) rabbit mAbs (#9664) and glyceraldehyde 3‐phosphate dehydrogenase (GAPDH) rabbit mAbs (#5174) were purchased from Cell Signalling Technology (Danvers, MA, USA). All antibodies were used at the dilution ratios recommended by the manufacturers.

### 2.2 Animals and Mouse Model

C57/BL6J mice were purchased from Spivey (Beijing) Biotechnology Co. Ltd. (Beijing, China), housed at the Animal Center of Central China Fuwai Hospital of Zhengzhou University with controlled temperature and humidity (temperature: 20°C–25°C; humidity: 50% ± 5%) and a dark: light cycle of 12:12 h and randomly given water and sterile food. All animal studies were performed in accordance with the Guidelines for Animal Care, approved by the Animal Care and Utilisation Committee of Central China Fuwai Hospital of Zhengzhou University (FZX‐IACUC‐2024004) and conducted in compliance with the National Institutes of Health Guide for the Care and Use of Laboratory Animals (NIH publication No. 85–23). After terminal studies at the indicated time points, the animals were euthanised using 3% isoflurane inhalation, followed by cervical dislocation.

### 2.3 DOX‐Induced Vascular Injury

C57/BL6J mice were administered DOX at a dose of 20 mg/kg via tail vein injection in a single dose, and seven days after DOX administration, the mice were euthanised. The entire aorta of the mouse was carefully separated using micro‐forceps and longitudinally dissected using microscissors, and the endothelial layer tissue (not including the smooth muscle layer tissue) was carefully removed for subsequent western blot analysis and quantitative polymerase chain reaction (qPCR) detection.

### 2.4 Cell Culture

Primary human umbilical vein endothelial cells (HUVECs) and human aortic endothelial cells (HAECs) were purchased from Sciencell (CA, USA) and cultured in endothelial cell‐specific medium (ECM) at 37°C in a 5% CO_2_ environment for use during generations 3–6. THP‐1 cells were purchased from ATCC (Manassas, VA, USA) and cultured in 1640 medium containing 10% foetal bovine serum (FBS) and 1% penicillin + streptomycin.

### 2.5 siRNA Transfection

The HUVECs were plated and cultured overnight. Small interfering RNAs (siRNAs) for knockdown of METTL3 and ISG15 were purchased from RiboBio Technologies (Guangzhou, China), and 50 nM siRNAs were co‐incubated with Lipofectamine RNAiMAX Transfection Reagent and then added to HUVECs. Cells were collected 48–72 h after transfection and subjected to the subsequent procedures. All siRNA sequences are listed in Table 1.

**TABLE 1:** Primers and siRNAs used in this study.

Primers for qPCR	Forward	Reverse
ISG15	GCAGATCACCCAGAAGATCG	TGCTGGTGGTGGACAAAT
ICAM‐1	GGGTAAGGTTCTTGCCCACT	TCCTCACCGTGTACTGGACT
VCAM‐1	GCCGAGCCAAATTACAAATTGATG	GAACAGGTCATGGTCACAGAGC
SELE	GCTCCAGGTGAACCCAACAA	TGGTACAGGCAGCTGTGTAG
**siRNAs**	**Target sequence**	
siMETTL3	5’‐CTGCAAGTATGTTCACTATGA‐3′	
si‐ISG15	5‐CTGAGCATCCTGGTGAGGAAT‐3′	
**meRIP‐qPCR Primers**	**Forward**	**Reverse**
ISG15‐1013516	CTGAGAGGCAGCGAACTCAT	CATGGCTGTGGGCTGTG
ISG15–1013869	CCTTCTAGTAACGAGCCCTCA	CAGGGCGAGCTCCTGTA
ISG15‐1013965	GAGCACTGTCCCTGGGT	CAGGCGTCACACAGGTT
ISG15‐1014147	CAGATCACCCAGAAGATCG	ATTTGTCCACCACCAGCA
ISG15‐1014220	TGCTGGTGGTGGACAAAT	TACCTCGTAGGTGCTGCT

### 2.6 Western Blot

Cells were extracted and homogenised in radioimmunoprecipitation assay (RIPA) buffer with pre‐added protease and phosphatase inhibitors and placed on ice for 30 min. The supernatant of the lysis products was collected, and the protein concentration was measured using the bicinchoninic acid (BCA) method. Next, 20–30 μg of the solubilised proteins were run on sodium dodecyl sulphate (SDS)‐polyacrylamide gel electrophoresis (PAGE) and transferred to nitrocellulose (NC) membranes, which were closed for 1 h at the end of the transfer using 5% skim milk. The membranes were then incubated with the primary antibody overnight at 4°C. On the next day, the primary antibody was washed off and the membranes were incubated with horseradish peroxidase‐labelled secondary antibody (Beyotime, China) for 1 h at room temperature. Next, the secondary antibody was washed off and the signals was detected using an ECL kit (Millipore, USA) and a chemiluminescence detection system (iBright, USA) and the intensity of the bands was analysed and quantified by ImageJ software.

### 2.7 Reverse Transcription‐qPCR

Total RNA was extracted from the cells using TRIzol reagent according to the manufacturer's instructions, and cDNA was synthesised using reverse transcriptase (Takara, Japan). Subsequent qPCR was performed using SYBR Green Premix Ex Taq (Takara, Japan) according to the manufacturer's instructions. All qPCR primers are listed in Table 1.

### 2.8 RNA Sequencing

HUVECs were transfected with NC or siMETTL3 for 48 h, and total cellular RNA was extracted using TRIzol reagent according to the manufacturer's instructions, after which genomic DNA was removed. RNA was quantified using a NanoDrop OneC, and the quality of the RNA was checked using a 2100 Bioanalyzer (Agilent). Sequencing libraries were constructed using RNA samples that passed the test, and bipartite libraries were synthesised using the TruSeq RNA Sample Preparation Kit (Illumina, USA), in accordance with the manufacturer's guidelines. After quantification using a Qubit 2.0 Fluorometer (Life Technologies, USA), sequencing was performed on an Illumina NovaSeq 6000. The results obtained from sequencing were assessed for quality using FastQC software. Differential expression analysis was performed using DESeq2, DEGseq and EdgeR. Genes showing |log2FC| > 1 and a Q value ≤ 0.05 according to DESeq2 and EdgeR, and those with a Q value ≤ 0.001 according to DEGseq were considered to be significant differentially expressed genes (DEGs).

### 2.9 iTRAQ Quantitative Proteomics

Cells were collected and lysed in protein lysate, sonicated and lysed on ice for 30 min and then centrifuged at 20,000 × *g* for 30 min at 4°C. The supernatants were then separated, and the protein supernatant concentrations were determined. Proteins were digested after quantification and labelled with different Itraq reagents for each group of peptides and analysed by on‐line electrospray tandem mass spectrometry on a Nano Aquity UPLC system (Waters Corporation) connected to an on‐line nanoelectrospray ionisation source equipped with an on‐line electrospray ionisation source (Michrom Bioresources). Proteins identified by two or more unique peptides were selected for further analyses. Differentially expressed proteins were selected based on the following criteria: (1) adjusted *p*‐value < 0.05 and (2) fold change ≥ 1.5.

### 2.10 Monocyte Adhesion Assay

HUVECs were transfected with the indicated siRNAs and then treated with DOX for 48 h. THP‐1 cells were pre‐labelled with 5‐(and 6)‐carboxyfluorescein diacetate and succinimidyl ester (10 mM) (MedChemExpress, Monuss Junction, NJ) for 30 min at 37°C. Labelled THP‐1 cells were co‐incubated with HUVECs for 30 min at 37°C and then washed two times with phosphate‐buffered saline (PBS) at the end of the incubation, adherent THP‐1 cells were photographed using inverted fluorescence microscopy and counted.

### 2.11 Methylated RNA Immunoprecipitation‐qPCR

Human METTL3 cDNA was generated by PCR and cloned into the pcDNA 3.1 expression plasmid (METTL3‐OE). Mut Express II Fast Mutagenesis Kit (Vazyme, #C214‐02) was used to generate the METTL3 catalytic mutant (aa395‐398, DPPW→APPA) (METTL3‐Mut) as described previously [14]. HUVECs were transfected with siRNAs (negative control small interfering RNA (siNC) or siMETTL3) and (or) plasmid (GFP, METTL3 overexpression or METTL3 methylation site mutation plasmid) for 48 h. Cells were harvested, and total RNA was extracted and fragmented using an RNA fragmentation kit (Thermo Fisher Scientific, AM8740), followed by purification of the fragmented RNA using ethanol precipitation. The fragmented RNA was mixed with methylated RNA immunoprecipitation (meRIP) buffer and incubated overnight at 4°C with protein A/G beads pre‐coated with IgG/m6A antibody. The next day, the samples were washed 3–4 times with high‐salt buffer and the RNA bound to the magnetic beads was extracted using TRIzol and subjected to subsequent qPCR assays.

### 2.12 Liquid Chromatography‐Mass Spectrometry

Total RNA was extracted from the cells, after which 500 ng of RNA was diluted with RNase‐free double‐distilled water (ddH_2_O) to a final volume of 10 μL; 0.3 U of nuclease P1 and 1 μL of 10× nuclease P1 buffer and 0.1 U of snake venom phosphodiesterase were added; the liquid at the bottom of the Eppendorf tubes was briefly centrifuged by mixing on a vortexer; and the tubes were incubated at 37°C for 2 h. After completion of incubation, 1 U of alkaline phosphatase and 1.3 μL of 10× alkaline phosphatase buffer were added, followed by incubation at 37°C for 60 min and the tubes were finally incubated at 75°C for 5 min to terminate the digestion. The processed samples were transported on dry ice for further analyses.

### 2.13 mRNA Stability Assay

Cells were pre‐plated and transfected with siRNA, and actinomycin D (2 μg/mL) was added to the culture set for 0, 1, 2, 3, 4, 5 or 6 h. Next, total cellular RNA was collected at each time point and reverse transcription (RT)‐qPCR was performed with GAPDH mRNA as the negative control.

### 2.14 Detection of Apoptosis by Flow Cytometry

Apoptosis detection was performed using the Annexin V‐Elab Fluor 647/PI Apoptosis Kit and carried out strictly according to the instructions. Briefly, HUVECs were cultured in 10‐cm dishes and apoptosis was induced using DOX. The cells were harvested using ethylenediaminetetraacetic acid (EDTA)‐free trypsin, washed twice with pre‐cooled PBS and resuspended in 100 μL of 1 × Annexin V Binding Buffer. Add 5 μL of Annexin V‐Elab Fluor 647 Reagent and 5 μL of PI Reagent (50 μg/mL) to the cell suspension. After gentle vortex mixing, incubate at room temperature in the dark for 15 to 20 min. At the end of the staining process, 400 μL of 1 × Annexin V Binding Buffer was added and detection was performed within 1 h using an LSRFortessa Flow Cytometer (BD Biosciences).

### 2.15 Luciferase Activity

For dual luciferase reporter gene assays, ISG15 mRNA sequences containing METTL3 methylation site (1014147) or mut were inserted into the pGL3‐promoter vector (Promega) to generate pGL3‐1,014,147‐WT or pGL3‐1,014,147‐Mut constructs. HUVECs were transfected with negative control (NC) or METTL3 (siMETTL3) siRNA with Lipofectamine 3000 (ThermoFisher Scientific, #L3000001). 48 h later, pGL3‐1,014,147‐WT or pGL3‐1,014,147‐Mut constructs were co‐transfected with the Renilla luciferase construct into these cells (NC or siMETTL3). Dual luciferase activities were then measured using the dual‐luciferase reporter gene system (Promega, #E1960) in accordance with the manufacturer's protocol. The relative luciferase activity was normalised to the Renilla luciferase internal control.

### 2.16 Statistical Analysis

The results were analysed using IBM SPSS Statistics software (version 23.0). Data are presented as mean ± SEM or SDs unless otherwise specified. One‐way analysis of variance (ANOVA) was used to determine the statistical significance for experiments with more than two groups, followed by Bonferroni's post hoc tests. Comparisons between the groups were performed using unpaired Student's *t*‐test. *P*‐values less than 0.05 were considered statistically significant.

## 3 Results

### 3.1 METTL3‐Mediated RNA Methylation is Involved in Doxorubicin‐Induced Endothelial Cell Apoptosis

**FIGURE 1:**
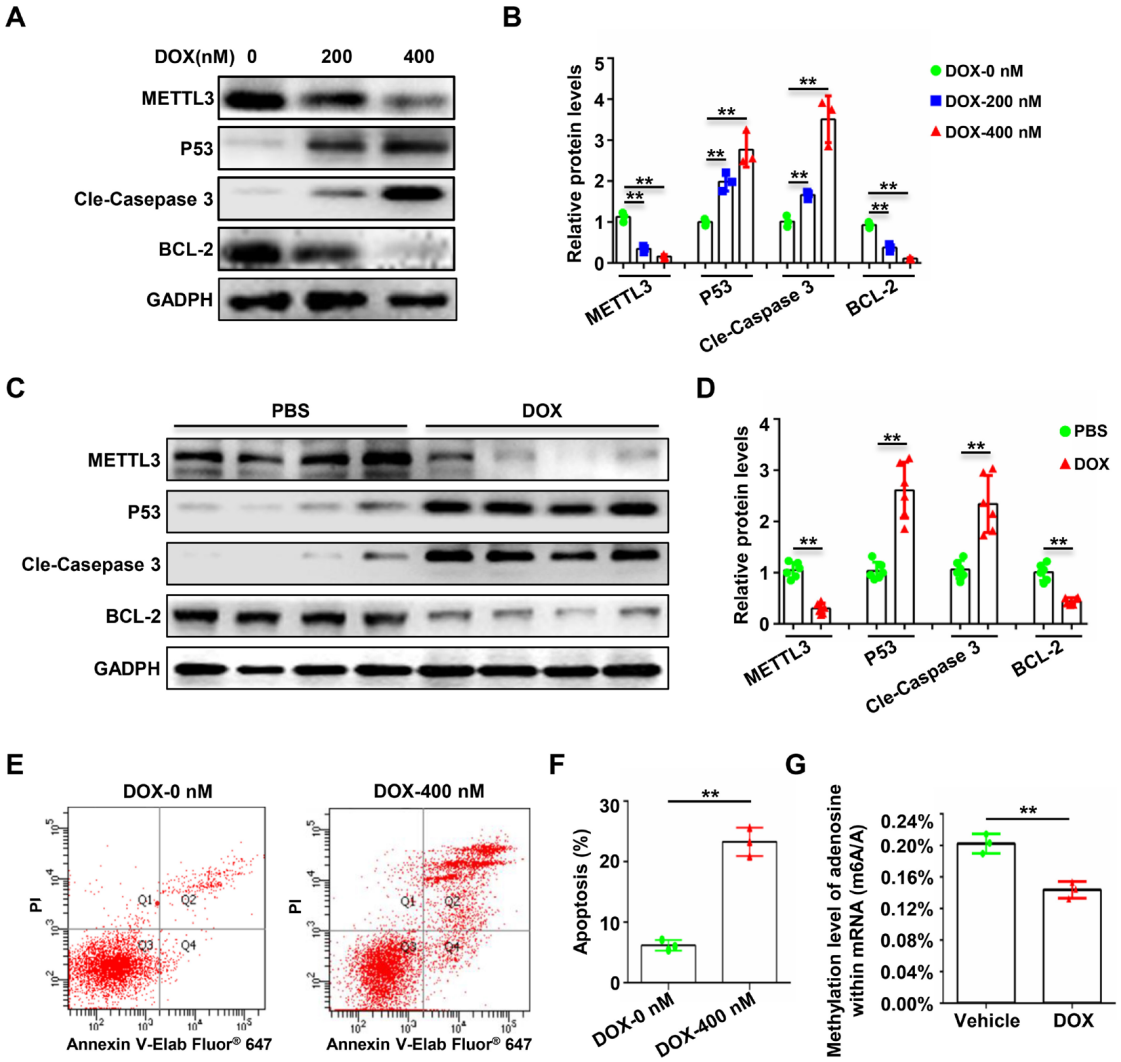
METTL3‐mediated RNA methylation is involved in chemotherapy‐induced endothelial cell apoptosis. (A and B) HUVECs were treated with DOX (0, 200 nM, or 400 nM) for 48 h to induce apoptosis. Immunoblotting analysis of the expressions of METTL3, P53, cleaved caspase 3, BCL‐2 and GAPDH (A) and the densities of the signals in (A) were scanned and plotted (B). Data were analysed by one‐way ANOVA followed by Bonferroni's post hoc tests and are presented as mean ± SEM from three independent experiments. **p < 0.01. (C) Mice were administered DOX (20 mg/kg) by tail vein injection to induce apoptosis of the vascular endothelium. Immunoblotting analysis of the expressions of METTL3, P53, cleaved caspase 3, BCL‐2 and GAPDH from the scraped endothelium from the two groups (*n* = 6 for both groups). (D) Densities of the blotting signals in (C) were scanned and plotted (*n* = 6 for each group). Data are presented as mean ± SD and were analysed by unpaired Student's *t*‐test. **p < 0.01. (E to G) HUVECs were treated with DOX (400 nM) for 48 h to induce apoptosis. Flow cytometry using Annexin V‐Elab Fluor 647/PI staining was performed (E) and the percentage of apoptotic cells was detected and analysed (F). (G) RNAs of cells from E were analysed by liquid chromatography‐mass spectrometry (LC–MS) and statistical tests. Data are presented as mean ± SD from three independent experiments and were analysed by unpaired Student's *t*‐test. * *p < 0.01.

To explore whether METTL3‐mediated RNA methylation is involved in chemotherapy drug‐related endothelial cell apoptosis, we first constructed a model of endothelial cell apoptosis induced by DOX, a classic chemotherapeutic drug commonly used clinically. As the concentration of DOX increased, the expression of pro‐apoptotic proteins in endothelial cells significantly increased, whereas that of anti‐apoptotic proteins significantly decreased. Concurrently, the expression of METTL3 significantly decreased with increasing DOX concentration (Figure 1A,B). Subsequently, we administered DOX via tail vein injections to mice to simulate the clinical chemotherapy process and then isolated endothelial cells to further confirm in vivo that DOX can induce apoptosis in endothelial cells and that METTL3 expression is significantly downregulated in this process (Figure 1C,D). In addition, to determine whether the reduction in METTL3 is accompanied by a decrease in RNA m6A modification during DOX‐induced endothelial cell apoptosis, we conducted liquid chromatography‐mass spectrometry analysis after inducing endothelial cell apoptosis with DOX and found that the overall m6A modification level of mRNA significantly decreased during DOX‐induced endothelial cell apoptosis (Figure 1E–G). These results suggest that METTL3‐mediated RNA methylation is involved in DOX‐induced endothelial cell apoptosis.

### 3.2 METTL3 Silencing Exacerbates DOX‐Induced Endothelial Cell Apoptosis

**FIGURE 2:**
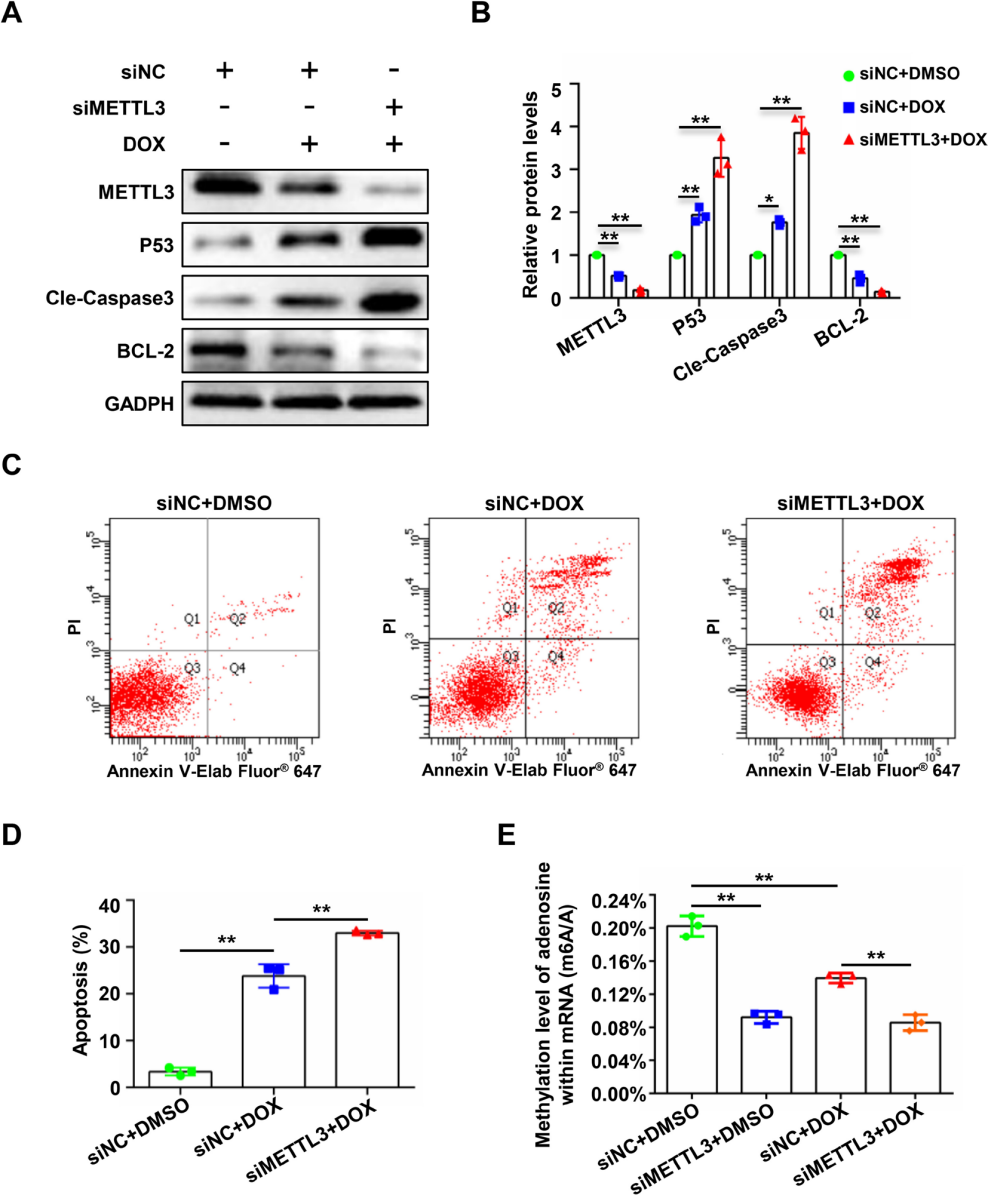
Silencing of METTL3 exacerbated doxorubicin‐induced apoptosis of HUVECs. HUVECs were divided into siNC, siNC + DOX and siMETTL3 + DOX groups and were transfected with NC siRNA (siNC) or siRNA‐METTL3 (siMETTL3). Apoptosis was induced by DOX (400 nM, 48 h). (A) Immunoblotting analysis of the expression of METTL3, P53, cleaved caspase 3, BCL‐2 and GAPDH in the three groups. (B) Densities of the blotting signals in (A) were scanned and plotted. Data were analysed by one‐way ANOVA followed by Bonferroni's post hoc tests and are presented as mean ± SEM from three independent experiments. *p < 0.05, **p < 0.01. (C) Flow cytometry using Annexin V‐Elab Fluor 647/PI staining was performed to analyse the apoptosis of the cells described in A. (D) Statistical analysis of the apoptosis rate in C. (E) RNAs of cells from the above groups were analysed by liquid chromatography‐mass spectrometry (LC–MS) and statistical tests. Data were analysed by one‐way ANOVA followed by Bonferroni's post hoc tests and are presented as mean ± SEM from three independent experiments. **p < 0.01.

To further explore the effect of METTL3 on endothelial cell apoptosis, first, we examined whether silencing METTL3 affects the apoptosis of endothelial cells in the absence of DOX induction. We found that silencing METTL3 did not affect the expression of apoptosis‐related proteins (Figure S1A,B). At the same time, flow cytometry also confirmed that silencing METTL3 did not increase the proportion of apoptotic cells (Figure S1C,D). Therefore, we believe that silencing METTL3 does not affect endothelial cell apoptosis under basal conditions. Furthermore, we silenced the expression of METTL3 in HUVECs and induced apoptosis using DOX. Western blotting results confirmed that in comparison with the control group, the groups in which METTL3 was silenced showed upregulated expression of pro‐apoptotic proteins and reduced expression of anti‐apoptotic proteins during DOX‐induced endothelial cell apoptosis (Figure 2A,B). Flow cytometry confirmed that silencing METTL3 before induction of apoptosis with DOX further increased the proportion of apoptotic cells (Figure 2C,D). Meanwhile, liquid chromatography‐mass spectrometry analysis confirmed that silencing METTL3 further decreased the overall m6A modification level of mRNA induced by DOX (Figure 2E). These results indicate that silencing METTL3 may further exacerbate DOX‐induced apoptosis in vascular endothelial cells in an m6A‐dependent manner.

### 3.3 Silencing METTL3 May Accelerate DOX‐Induced Endothelial Cell Apoptosis by Promoting the Expression of ISG15

**FIGURE 3:**
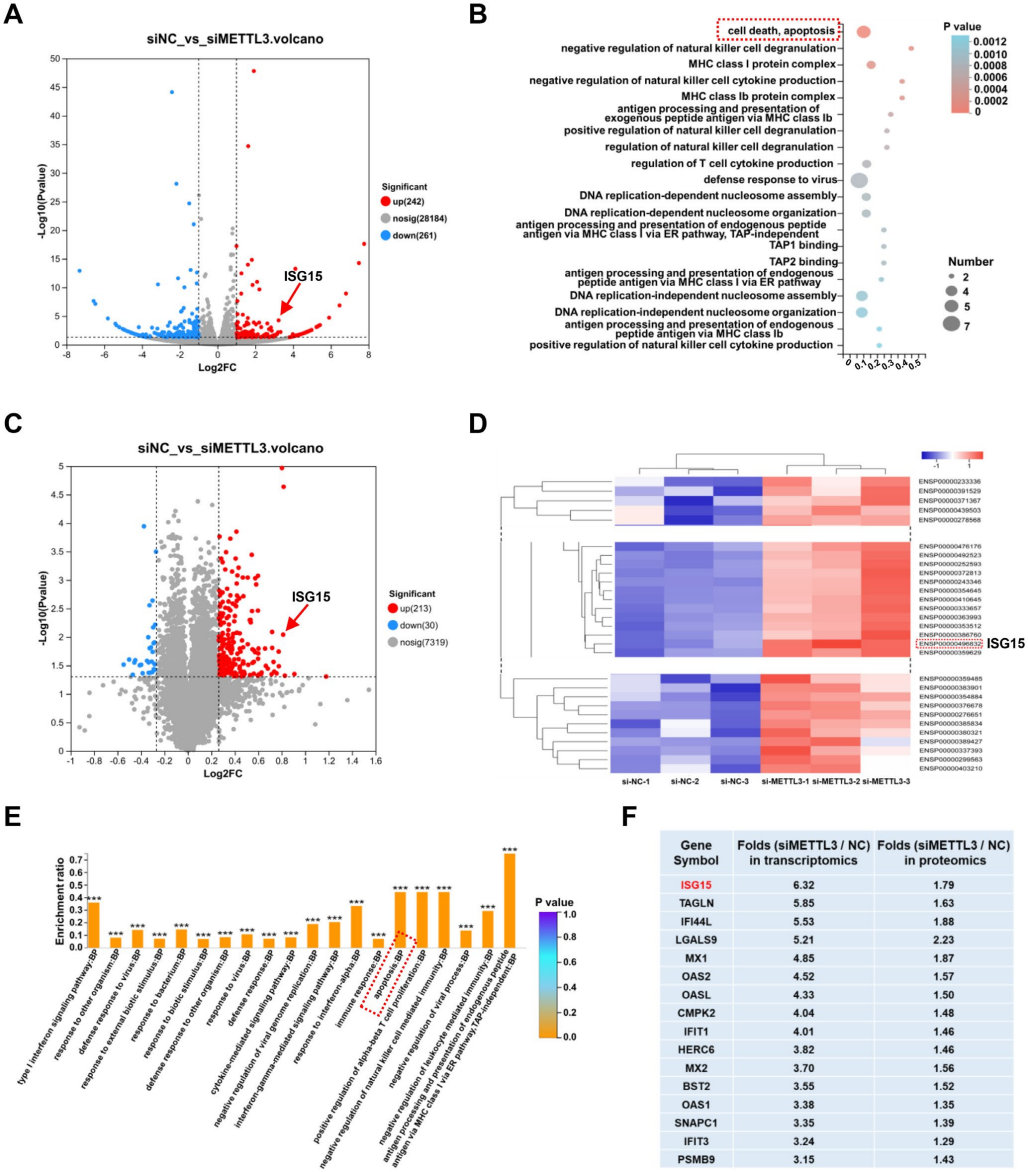
Proteomics and transcriptomics analyses indicated that METTL3 may regulate endothelial cell apoptosis by regulating the expression of ISG15. HUVECs were transfected with NC siRNA (siNC) or siRNA‐METTL3 (siMETTL3) for 48 h. The mRNA and protein samples were collected for subsequent analyses. (A) Volcano plot of differentially regulated mRNA transcripts in HUVECs transfected with NC siRNA in comparison with those transfected with siMETTL3. ISG15 is indicated by the red arrow. (B) Gene ontology (GO) enrichment analysis of genes showing altered expression between the siNC and siMETTL3 groups, as determined by transcriptomic analysis. (C) Volcano plot showing the changes in protein expression after HUVECs were transfected with NC siRNA in comparison with those transfected with siMETTL3. ISG15 is indicated by the red arrow. (D) Heatmap showing differentially expressed proteins between the NC siRNA‐ and siMETTL3‐transfected groups identified by proteomics analysis. ISG15 is indicated by the red dashed rectangle. (E) GO enrichment analysis of differentially expressed proteins in the proteome. BP: Biological Process. (F) Transcriptome‐proteome joint analysis of genes that were significantly elevated at both the transcriptional and protein levels in the NC siRNA‐ and siMETTL3‐transfected groups.

To elucidate the detailed mechanism through which METTL3 regulates apoptosis in vascular endothelial cells, we silenced the expression of METTL3 in HUVECs and performed proteomics and transcriptomics analyses. The results indicated that METTL3 silencing was followed by a significant change in the expression of genes related to the vascular endothelium (Figure 3A) and gene ontology (GO) analysis showed that genes related to apoptosis pathways were significantly upregulated (Figure 3B). Proteomics analysis also indicated that after silencing METTL3, the expression levels of genes related to apoptosis pathways were significantly upregulated (Figure 3C–E). Combined transcriptomics and proteomics analyses suggested that the expression of ISG15 was significantly upregulated at both the transcriptional and protein levels (Figure 3F).

ISG15, an important interferon‐induced protein, is rapidly and strongly expressed under interferon stimulation and plays a role in antiviral regulation [15]. Previous studies have confirmed that ISG15 plays a significant role in apoptosis [16]. Therefore, based on the aforementioned experimental results and previous studies on the apoptotic regulatory role of ISG15, we speculated that METTL3 may regulate endothelial cell apoptosis by modulating the expression of ISG15.

### 3.4 METTL3 Directly Methylates ISG15 mRNA and Inhibits its Expression

**FIGURE 4:**
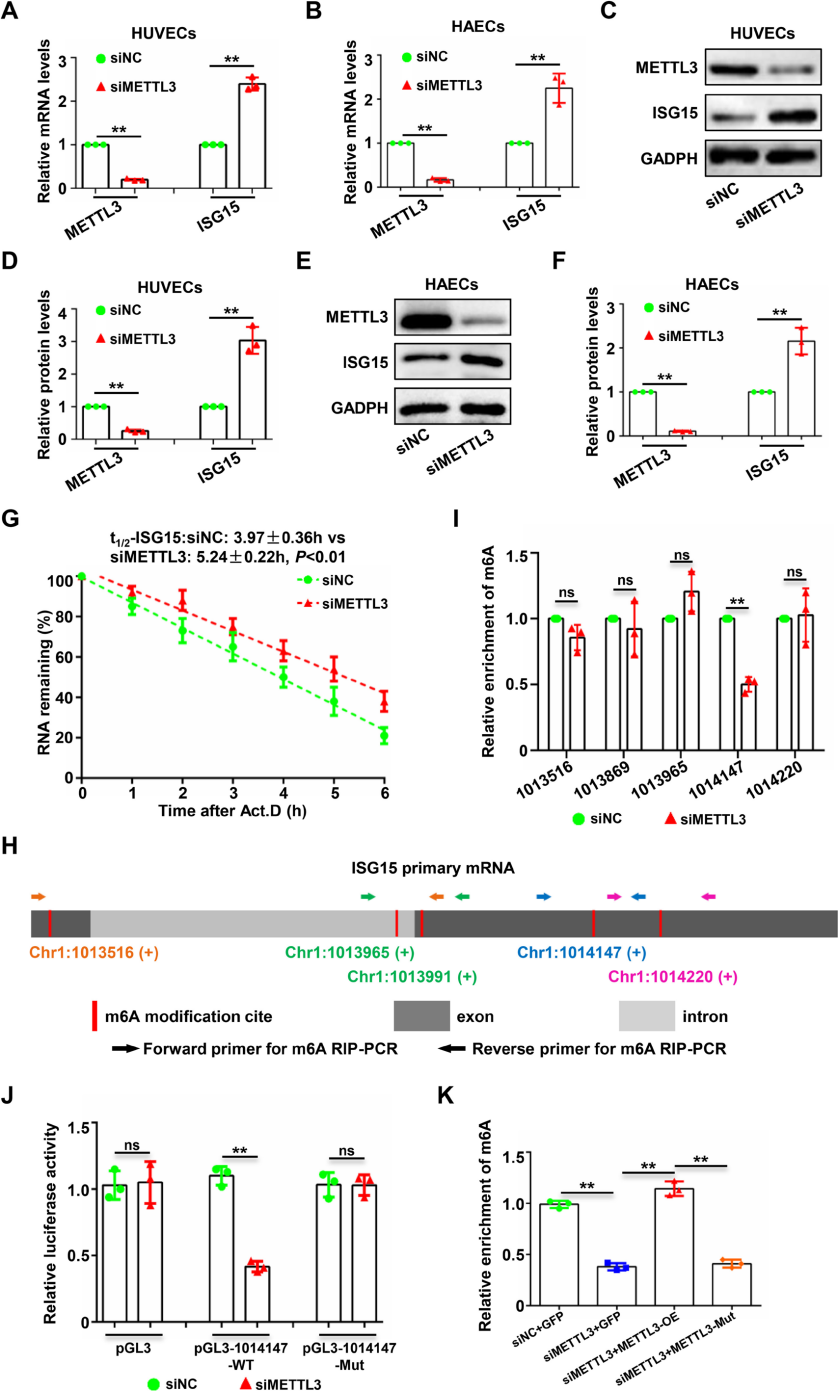
METTL3 can directly methylate ISG15 mRNA. HUVECs or HAECs were transfected with NC siRNA (siNC) or siRNA‐METTL3 (siMETTL3) for 48 h. The mRNA and protein samples were collected for subsequent analyses. (A and B) The mRNA expression levels of ISG15 and METTL3 were detected using qPCR and statistical analysis. (C to F) Immunoblotting analysis of METTL3 and ISG15 expression (C and E), and the densities of the blotting signals were scanned and plotted (D and F). (G) HUVECs were transfected with siNC or siMETTL3 for 48 h followed by induction with actinomycin D at the indicated time points. The remaining ISG15 mRNA levels were detected by qPCR at different time points. (H) Schematic diagram of potential m6A modification sites on ISG15 mRNA predicted using bioinformatics and the corresponding meRIP‐qPCR primer design. (I) meRIP‐qPCR was performed using the indicated primers after HUVECs were transfected with siNC or siMETTL3 for 48 h. (J) Dual luciferase activity analysis of the generated reporter constructs within HUVECs receiving METTL3 siRNA or not. (K) meRIP‐qPCR was performed using the 1,014,147 site specific primers of ISG15 mRNA after HUVECs were transfected with siNC, siMETTL3 and (or) METTL3 overexpression plasmid (METTL3‐OE) or METTL3 catalytic mutant plasmid (METTL3‐Mut) for 48 h. Data are presented as mean ± SD from three independent experiments and were analysed by unpaired Student's *t*‐test. ns, no significance; **p < 0.01.

To explore the detailed molecular mechanisms by which METTL3 regulates ISG15 expression, we silenced METTL3 in two different endothelial cells (HUVECs and HAECs) and measured ISG15 protein and mRNA expression levels. The results showed that after silencing METTL3, the expression levels of both ISG15 mRNA (Figure 4A,B) and protein (Figure 4C–F) were significantly upregulated. Thus, METTL3 may regulate the expression of ISG15 by affecting the half‐life of ISG15 mRNA. Furthermore, we conducted RNA‐degradation experiments in which endothelial cells were treated with actinomycin D while METTL3 was silenced. Silencing METTL3 significantly prolonged the half‐life of ISG15 mRNA (Figure 4G). To further clarify the specific sites of METTL3 methylation on ISG15 mRNA, we first predicted all potential m6A methylation sites on ISG15 primary mRNA using bioinformatics and identified a total of five potential binding sites (Figure 4H). Then, we designed qPCR primers for these five sites and conducted meRIP‐qPCR assays to determine the binding sites for METTL3‐mediated ISG15 mRNA methylation. Our results showed that the 101417th site in the ISG15 mRNA exon may be the site of m6A modification mediated by METTL3 (Figure 4I). To determine whether METTL3 promotes ISG15 expression via RNA methylation, we generated reporter genes bearing ISG15 RNA methylation regions (1,014,147 sites) or not and these reporter constructs were separately transfected into HUVECs treated with control or METTL3 siRNA. Our data showed that METTL3 knockdown significantly decreased the luciferase activity in cells transfected with the pGL3‐1,014,147‐WT construct but had no influence on that in cells transfected with the pGL3 or pGL3‐1,014,147‐Mut construct (Figure 4J), attesting to the dependence on RNA methylation for ISG15 regulation by METTL3. Finally, to confirm METTL3 is sufficient to methylate ISG15 mRNA, we silenced METTL3 in HUVECs while transfecting lentiviruses overexpressing METTL3 or with METTL3 methylation site mutations and then conducted meRIP experiments. We found that overexpression of METTL3 could rescue the reduction in ISG15 mRNA binding to m6A caused by METTL3 silencing, whereas transfection with lentiviruses carrying METTL3 methylation site mutations did not have this effect (Figure 4K). This suggests that the methylation of ISG15 mRNA indeed depends on METTL3. Based on these findings, we believe that METTL3 promotes ISG15 mRNA degradation by directly methylating it, thereby inhibiting its expression.

### 3.5 Silencing of ISG15 Significantly Inhibits the DOX‐Induced Endothelial Cell Apoptosis Exacerbated by Silencing METTL3

**FIGURE 5:**
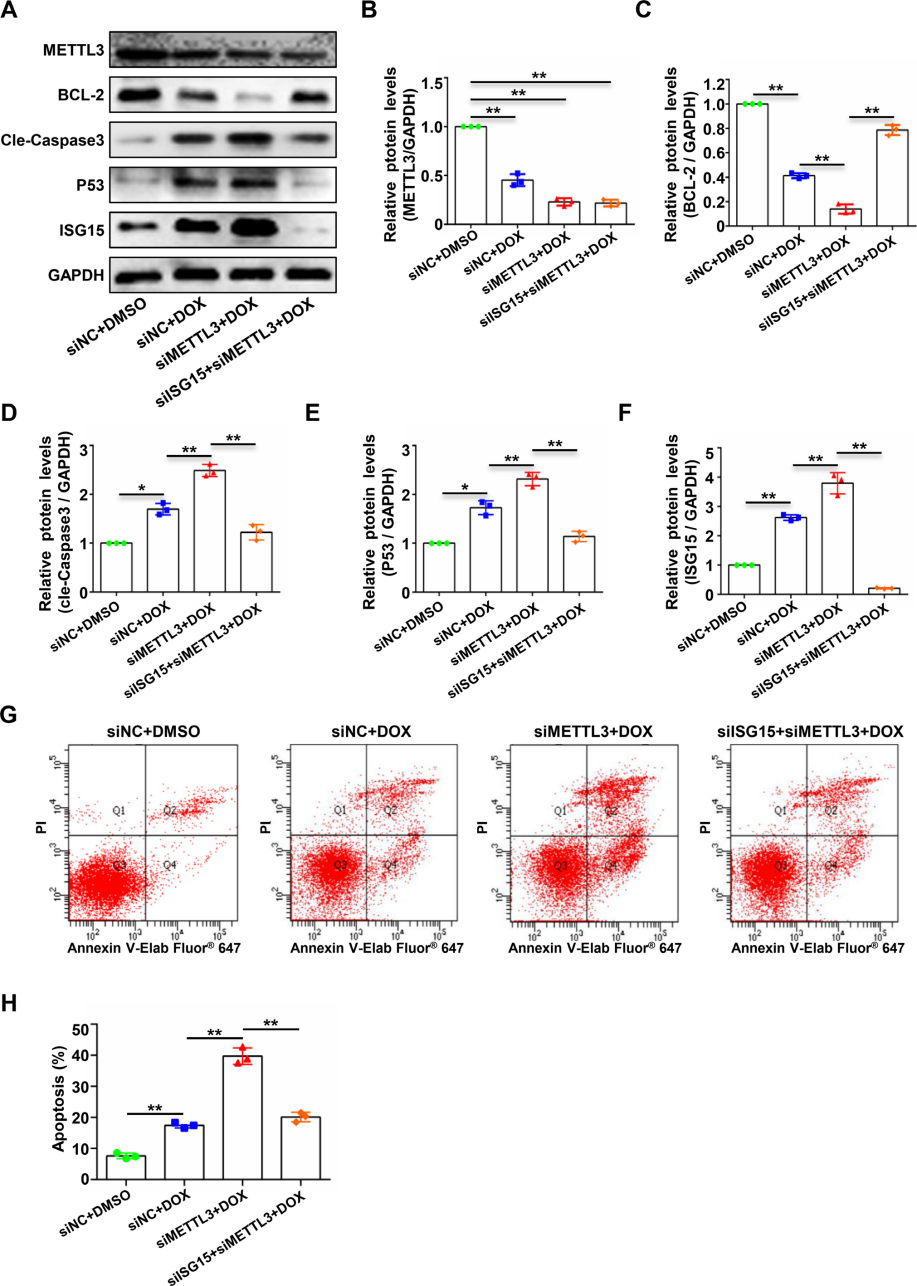
Silencing of ISG15 significantly inhibits the doxorubicin‐induced endothelial cell apoptosis exacerbated by silencing METTL3. HUVECS were divided into siNC, siNC + DOX, siMETTL3 + DOX and siMETTL3 + siISG15 + DOX groups and transfected with NC siRNA (siNC), siRNA‐METTL3 (siMETTL3), or (and) siRNA‐ISG15 (siISG15) and apoptosis was induced by DOX (400 nM, 48 h). (A to F) Immunoblotting analysis of the expression of METTL3, ISG15, and the apoptosis‐related proteins cleaved caspase 3, BCL‐2 and P53 (A); the densities of the blot signals from A were scanned and plotted (B to F). (G and H) Flow cytometry using Annexin V‐Elab Fluor 647/PI staining was performed to analyse the apoptotic cells from the above four groups (C), and the percentage of apoptotic cells in each group was statistically analysed (D). Data were analysed by one‐way ANOVA followed by Bonferroni's post hoc tests and are presented as mean ± SEM from three independent experiments. *p < 0.05, **p < 0.01.

To explore whether METTL3 regulates DOX‐induced endothelial cell apoptosis by modulating the expression of ISG15, we silenced both METTL3 and ISG15 in a DOX‐induced endothelial cell apoptosis model. Silencing METTL3 exacerbated the apoptosis of endothelial cells during DOX‐induced endothelial cell apoptosis, an effect that could be mitigated by silencing the expression of ISG15 (Figure 5A–F). Concurrently, flow cytometry assays confirmed that silencing ISG15 expression after METTL3 silencing significantly reduced DOX‐induced endothelial cell apoptosis rate (Figure 5G,H). Our findings confirmed that METTL3 participates in DOX‐induced endothelial cell apoptosis by regulating the expression of ISG15.

### 3.6 Inhibition of ISG15 can Significantly Alleviate the Secretion of Endothelial Inflammatory Factors and Monocyte‐Endothelial Adhesion Mediated by METTL3 Silencing

**FIGURE 6:**
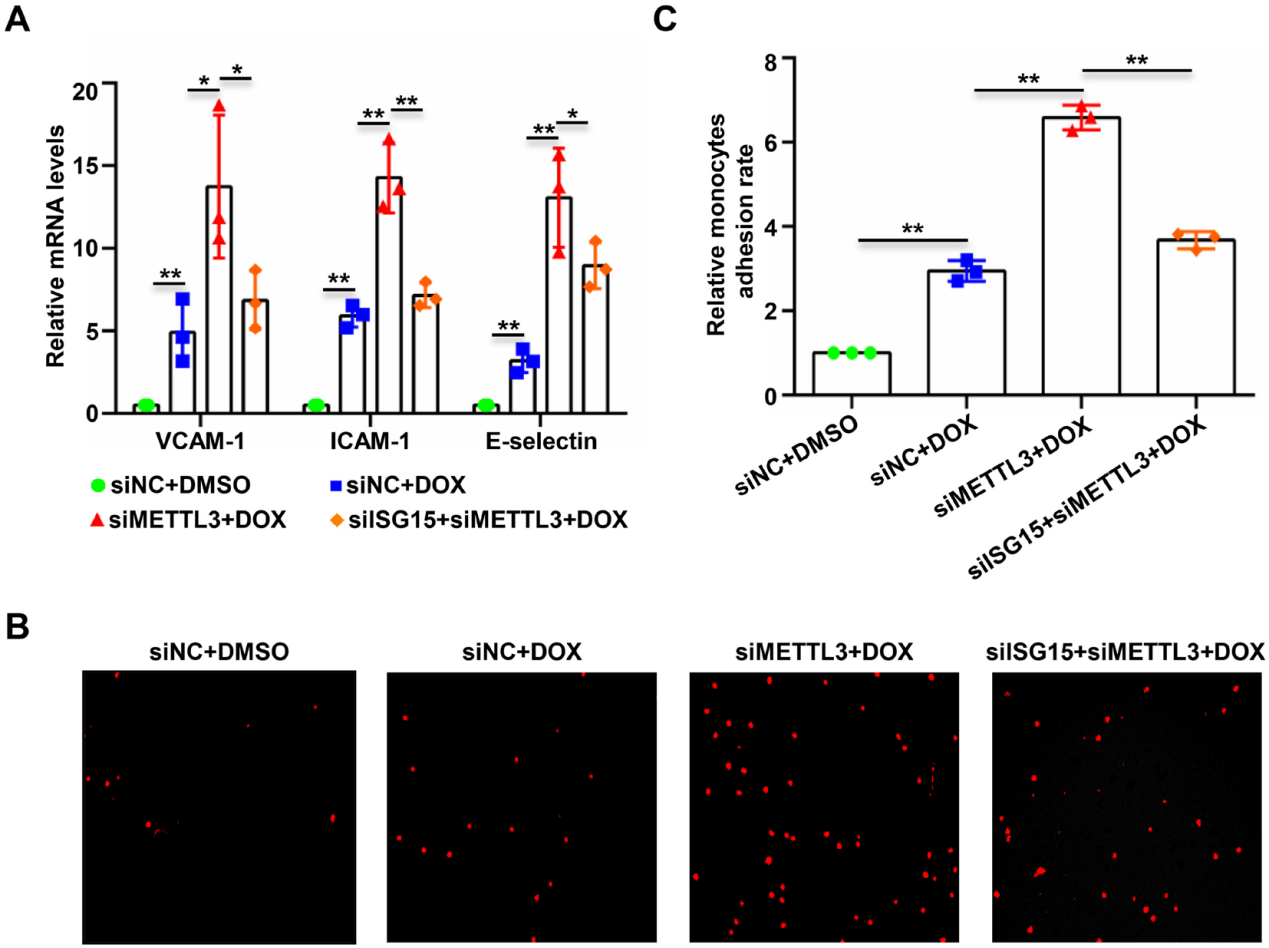
Inhibition of ISG15 can significantly alleviate the secretion of endothelial inflammatory factors and monocyte‐endothelial adhesion mediated by METTL3 silencing. HUVECS were divided into siNC, siNC + DOX, siMETTL3 + DOX and siMETTL3 + siISG15 + DOX groups and transfected with NC siRNA (siNC), siRNA‐METTL3 (siMETTL3) or siRNA‐ISG15 (siISG15) and apoptosis was induced by DOX (400 nM, 48 h). (A) The mRNA expression levels of VCAM‐1, ICAM‐1 and E‐selectin were detected by qPCR and statistical analysis. (B and C) Monocyte‐endothelial adhesion assay was performed in the above four groups, and the adherent THP‐1 cells were photographed using inverted fluorescence microscopy (B) and the relative adhesion rates were statistically analysed. Data were analysed by one‐way ANOVA followed by Bonferroni's post hoc tests and are presented as mean ± SEM from three independent experiments. *p < 0.05, **p < 0.01.

To further clarify the effect of METTL3 regulation of ISG15 expression on chemotherapy‐related endothelial function, we silenced ISG15 expression while silencing METTL3. We then measured the mRNA expression of inflammatory factors such as VCAM‐1, ICAM‐1 and E‐selectin. Our results suggest that silencing METTL3 significantly promoted the expression of DOX‐induced endothelial cell inflammatory factors, and silencing ISG15 significantly suppressed the upregulation of inflammatory factors induced by the knockdown of METTL3 (Figure 6A). Concurrently, after silencing ISG15 expression while silencing METTL3, we induced endothelial cell apoptosis with DOX and performed monocyte‐endothelial adhesion experiments. The results indicated that silencing METTL3 significantly enhanced DOX‐induced monocyte‐endothelial adhesion, and silencing ISG15 significantly alleviated the upregulation of monocyte‐endothelial adhesion caused by METTL3 silencing (Figure 6B,C). The aforementioned experimental results demonstrated that inhibiting ISG15 can significantly alleviate the secretion of endothelial inflammatory factors and monocyte‐endothelial adhesion mediated by METTL3 silencing.

## 4 Discussion

METTL3 is a key enzyme responsible for methyl transfer in the m6A modification process and participates in the regulation of various biological processes [17, 18]. An increasing number of studies have shown that METTL3 plays an important regulatory role in the process of apoptosis. For example, METTL3 can regulate chondrocyte apoptosis and autophagy in temporomandibular joint arthritis by regulating the m6A/YTHDF1/Bcl‐2 signalling axis [9]. The m6A modification mediated by METTL3 can also affect the proliferation and apoptosis of lens epithelial cells in diabetic cataracts [19]. METTL3 silencing attenuates high glucose‐induced vascular endothelial cell apoptosis by promoting SOCS3 stability [20]. In this study, we found that METTL3 participates in the DOX‐induced apoptosis of endothelial cells by directly methylating ISG15 mRNA. This suggests that METTL3 is a potential target for intervention in chemotherapy‐related apoptotic processes.

A large number of studies have confirmed that various stimuli, including DNA damage, radiation, telomere shortening and lipopolysaccharide treatment, can induce the expression of ISG15, thereby regulating biological processes such as cell apoptosis, ageing, autophagy and the immune response [15]. In addition, during doxorubicin‐induced DNA damage in tumour cells, ISG15 can enhance the transcriptional activation of downstream apoptosis‐related target genes, such as p21 and Bcl‐2‐like protein 4 (BAX), by ubiquitination of p53, promoting cell apoptosis. At the same time, during this process, the expression of ISG15 depends on the transcriptional activation of p53, thus forming a positive feedback regulatory loop to enhance the cell growth inhibition and apoptosis induced by DOX [21]. We further predicted the m6A modification sites of ISG15 RNA and verified them through meRIP‐qPCR; we found that METTL3 can directly methylate ISG15 mRNA and that the 1014147th site of the ISG15 mRNA exon may be the m6A modification site mediated by METTL3, thereby regulating the expression of ISG15. Previous studies have reported that METTL3 indirectly regulates the expression of interferon‐induced proteins, including ISG15, by regulating the IFN‐JAK/STAT pathway [22]. In this study, we found that METTL3 methylates ISG15 mRNA to directly regulate the expression of ISG15, affecting DOX‐induced endothelial cell apoptosis. Thus, our study deepens the understanding of the regulatory mechanisms of METTL3 in apoptosis.

This study focused on endothelial cell apoptosis induced by chemotherapy drugs, and through a series of in vivo and in vitro experiments, it preliminarily elucidated the regulatory effects of METTL3‐mediated ISG15 mRNA methylation on endothelial cell apoptosis as well as the impact of these regulatory effects on the expression of endothelial inflammatory‐related factors and monocyte‐endothelial adhesion, providing new ideas for the protection of endothelial cells in patients receiving chemotherapy during clinical diagnosis and treatment. Further research is needed to clarify the regulation of METTL3‐mediated ISG15 mRNA methylation in endothelial cell apoptosis in endothelial cell‐specific METTL3 knockout mice or human samples and to explore its impact on endothelial cell function.

## Author Contributions

**Dongdong Jian:** conceptualization (equal), formal analysis (equal), funding acquisition (equal), investigation (equal), methodology (equal), supervision (equal), writing – review and editing (equal). **Han Li:** investigation (equal). **Chenqiu Wang:** formal analysis (equal). **Fang Li:** investigation (equal), validation (equal). **Runhua Li:** investigation (equal), validation (equal). **Shouyi Jin:** investigation (equal), validation (equal). **Jia Shen:** investigation (equal), validation (equal). **Jiamian Chen:** formal analysis (equal). **Wanjun Zhang:** formal analysis (equal). **Ling Pan:** investigation (equal), validation (equal). **Wengong Wang:** data curation (equal), project administration (equal). **Hao Tang:** data curation (equal), project administration (equal). **Liguo Jian:** conceptualization (equal), funding acquisition (equal), investigation (equal), supervision (equal). **Datun Qi:** conceptualization (equal), methodology (equal), supervision (equal).

## Conflicts of Interest

The authors declare no conflicts of interest.

## Supporting information


**Figure S1.** HUVECs were transfected with NC siRNA (siNC) or siRNA‐METTL3 (siMETTL3) for 48 h. (A) Immunoblotting analysis of the expression of METTL3, P53, cleaved caspase 3, BCL‐2 and GAPDH. (B) Densities of the blotting signals in (A) were scanned and plotted. (C) Flow cytometry using Annexin V‐Elab Fluor 647/PI staining was performed to analyse the apoptosis of the cells. (D) Statistical analysis of the apoptosis rate in C. Data are presented as mean ± SD from three independent experiments and were analysed by unpaired Student’s *t*‐test. ns, no significance; ** p < 0.01.

## Data Availability

The data and study materials will be available upon reasonable request.
