# Supplementary figures and images for: METTL3‐Mediated m6A Modification of ISG15 mRNA Regulates Doxorubicin‐Induced Endothelial Cell Apoptosis
Source: J Cell Mol Med. 2025 Jan 9;29(1):e70339. doi: 10.1111/jcmm.70339 (PMC11717669; doi:10.1111/jcmm.70339)

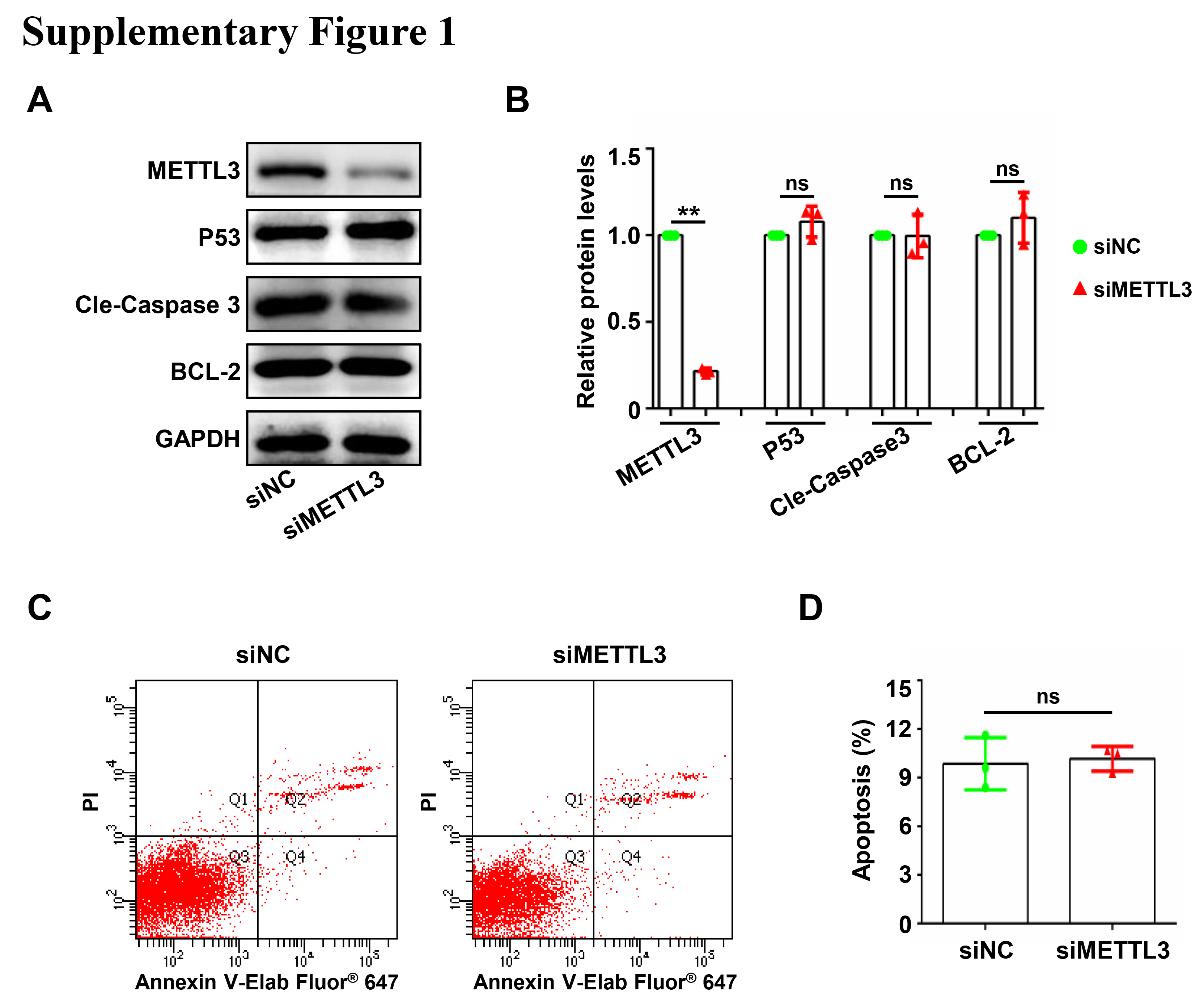

Supplement: Supplementary file 1 — Figure S1. HUVECs were transfected with NC siRNA (siNC) or siRNA‐METTL3 (siMETTL3) for 48 h. (A) Immunoblotting analysis of the expression of METTL3, P53, cleaved caspase 3, BCL‐2 and GAPDH. (B) Densities of the blotting signals in (A) were scanned and plotted. (C) Flow cytometry using Annexin V‐Elab Fluor 647/PI staining was performed to analyse the apoptosis of the cells. (D) Statistical analysis of the apoptosis rate in C. Data are presented as mean ± SD from three independent experiments and were analysed by unpaired Student’s t‐test. ns, no significance; ** p < 0.01. [file JCMM-29-e70339-s001.tif]
